# Mechanism of drug-induced liver injury and hepatoprotective effects of natural drugs

**DOI:** 10.1186/s13020-021-00543-x

**Published:** 2021-12-11

**Authors:** Yongfeng Zhou, Junnan Wang, Dingkun Zhang, Jiaxin Liu, Qinghua Wu, Jiang Chen, Peng Tan, Boyu Xing, Yanzhong Han, Ping Zhang, Xiaohe Xiao, Jin Pei

**Affiliations:** 1 College of Pharmacy, Chengdu University of Traditional Chinese Medicine, 1166 Liutai Avenue, Wenjiang District, Chengdu, 611137 Sichuan China; 2 School of Chinese Pharmacy, Beijing University of Chinese Medicine, Beijing, 102488 China; 3 Department of Pharmacy, Medical Supplies Center of PLA General Hospital, 100#, West 4th Ring Middle Rd., Fengtai, Beijing, 10039 China; 4 Department of Liver Disease, Fifth Medical Center of PLA General Hospital, 100#, West 4th Ring Middle Rd., Fengtai, Beijing, 10039 China

**Keywords:** Natural medicines, Drug-induced liver injury, Bioactive components, Mechanism

## Abstract

Drug-induced liver injury (DILI) is a common adverse drug reaction (ADR) and a serious threat to health that affects disease treatments. At present, no targeted clinical drugs are available for DILI. Traditional natural medicines have been widely used as health products. Some natural medicines exert specific hepatoprotective effects, with few side effects and significant clinical efficacy. Thus, natural medicines may be a promising direction for DILI treatment. In this review, we summarize the current knowledge, common drugs and mechanisms of DILI, as well as the clinical trials of natural drugs and their bioactive components in anticipation of the future development of potential hepatoprotective drugs.

## Introduction

Drug-induced liver injury (DILI) is an adverse drug reaction (ADR) that occurs in clinical applications. With the aggravation of the disease, DILI may progress to liver fibrosis, liver failure and even death and is a serious health threat [1, 2]. According to incomplete statistics, more than 1000 drugs may cause different degrees of liver injury [3]. In Western countries, the incidence of DILI is estimated to be (1–20)/100,000 of the total population [4–7]. The incidence of DILI in the state of Delaware, United States, is 2.7/100,000 [8]. In China, the annual incidence in the general population is 23.80/100,000, which is higher than that reported in Western countries. Traditional Chinese medicine and dietary supplements (26.81%) and anti-tuberculosis drugs (21.99%) are the two main categories of drugs causing DILI [9].

Some commonly used clinical drugs for DILI treatment include tiopronin, polyene phosphatidylcholine, ursodeoxycholic acid and N-acetylcysteine [10]. However, because the mechanism underlying the development of DILI is multisourced and complex, specific drugs for its treatment are still lacking.

Natural medicine is a treasure from nature to humans. Some natural drugs exert significant hepatoprotective effects on individuals with DILI. The concept of ‘natural therapy’ has been proposed in recent years, causing more people to pay attention to natural drugs. Most natural drugs have some problems, such as unclear compositions or unclear mechanisms of action, which seriously hinder their clinical applications and the development of new drugs.

In this paper, we performed a literature search using mainly Web of Science, PubMed, Google Scholar, CNKI, and VIP. We summarize the common types of drugs that cause DILI, the causes of liver injury, the mechanism of liver protection, the material basis of natural drugs and the clinical application of related drugs to provide a scientific basis and corresponding countermeasures for the development of potential hepatoprotective drugs based on natural medicine.

## Common drugs and mechanism of DILI

### Anti-TB drugs

Antituberculosis DILI (ATDILI) is a common form of DILI that often leads to the interruption of tuberculosis treatment and hinders the treatment process. At present, widely used anti-tuberculosis drugs in clinical applications with significant effects are mainly isoniazid (INH), rifampicin (RFP), aminosalicylic acid and levofloxacin, which can lead to DILI. As the applied doses of these drugs increase, their adverse reactions become more obvious [11]. Some factors, including female, drinking alcohol, liver disease, systemic lupus erythematosus or malnutrition, also increase the probability of liver damage caused by antituberculosis drugs [12, 13]. In recent years, researchers have found that CYP450, UGT, GST, NAT and other drug-metabolizing enzymes, as well as ABCB1, ABCC2 and other drug transporters and human leukocyte antigen gene polymorphisms, are positively correlated with the incidence of ATDILI [14, 15]. Furthermore, a polymorphism in the cytochrome P450 oxidoreductase gene, rs3898649, is significantly associated with ATDILI susceptibility, suggesting that it may be a potential biomarker involved in ATDILI [16] and could be helpful in the clinical diagnosis of ATDILI.

**Fig. 1:**
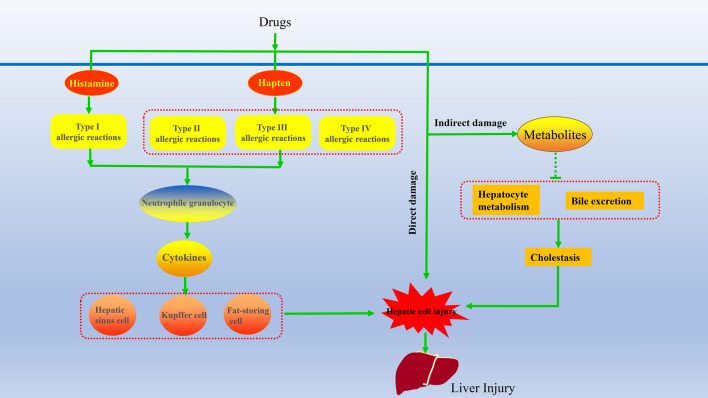
Possible mechanism of Anti-TB drugs (example as INH). Drugs can make hepatic cells continuously release inflammatory cytokines and response-induced stress kills hepatocytes. Direct hepatotoxicity of drugs and their metabolites includes the deformation and necrosis of hepatocytes

At present, the occurrence of ATDILI is presumed to mainly involve immune mechanisms and nonimmune mechanisms [17]. The immune mechanism is based on the increase in the histamine content in the body and induction of type I allergies by antituberculosis drugs. They can also use haptens to form an immune complex that causes type II, III and IV allergies and subsequent liver injury. The immune response recruits inflammatory cells that continuously release cytokines to act on hepatic sinusoidal cells, resulting in local circulatory disorders. Inflammatory response-induced stress kills hepatocytes. This process has no relation with the dosage. Low doses of rifampicin, isoniazid and pyrazinamide cause immune-mediated liver injury [18]. The nonimmune mechanism mainly involves the metabolic reaction of anti-TB drugs in vivo. Two main types of metabolic damage have been identified: direct and indirect damage to hepatocytes. However, both types have an obvious dose–response relationship with the drug dose, such as isoniazid, aminothiourea, rifampicin, ethylthioisoniazid, pyrazinamide and isonicotinamide. Direct hepatotoxicity of drugs and their metabolites includes the deformation and necrosis of hepatocytes. In addition, metabolites and drugs inhibit or block the metabolic function of hepatocytes or bile excretion function to subsequently damage hepatocytes or disrupt bile excretion, resulting in liver injury [19] (Fig. 1).

### Antitumour drugs

Antitumor drugs exert a therapeutic effect by inhibiting or killing tumor cells in the body. Thus, they inevitably damage normal cells, tissues and organs, which may lead to many adverse reactions, including DILI and kidney injury. Liver injury caused by these drugs usually occurs 1–2 weeks after medication and may result in pathological symptoms, such as hepatocyte necrosis, cholestasis and mixed liver injury. The combination of multiple antitumor drugs increases the incidence of liver injury [20]. At present, research on platinum antitumor drugs is relatively extensive. According to reports, oral antitumor drugs are the main mode of administration, accounting for 30–50% of all antitumor therapies. Oral administration improves the safety of tumor treatment, but in some cases, the administration of an improper causes adverse effects [21]. The appropriate adjustment of the drug dosage based on hepatotoxicity examinations might effectively prevent liver damage caused by antitumor drugs [22].

Cisplatin (CP) is the first platinum antitumor drug approved by the U.S. Food and Drug Administration (FDA). It has a wide antibacterial spectrum, strong antitumor effect and antitumor activity greater than 60%. It is a first-line chemotherapeutic drug [23]. However, CP significantly reduces immune function and causes liver injury while resisting tumor cells [24]. CP is metabolized in the liver, and its accumulation in the liver is second only to that in the kidney. Its injury is mainly caused by inducing oxidative stress, apoptosis and inflammatory injury in the liver. Reactive oxygen species (ROS) accumulation following CP-induced ROS production or abnormal expression of cytochrome P450 2E1 (CYP2E1) results in oxidative stress and injury in the liver [25].

### Nonsteroidal anti-inflammatory drugs

Nonsteroidal anti-inflammatory drugs (NSAIDs) have good clinical analgesic and antipyretic effects. However, liver injuries lead to cancellation or withdrawal from the market during the premarket or postmarket evaluation stage. Seven types of NSAIDs cause severe DILI, including diclofenac, ibuprofen, sulindac, aspirin, naproxen, piroxicam and nimesulide. These drugs account for 99% of all NSAIDs [26].

Diclofenac is an NSAID that causes rare but severe liver toxicity through an unclear mechanism. Some studies have explored the role of the immune response in the process of liver injury. By determining the genetic polymorphisms in patients, researchers found that low levels of IL-10 and high levels of IL-4 gene transcription contribute to Th-2-mediated antibody binding to new antigens related to disease susceptibility [27].

**Fig. 2:**
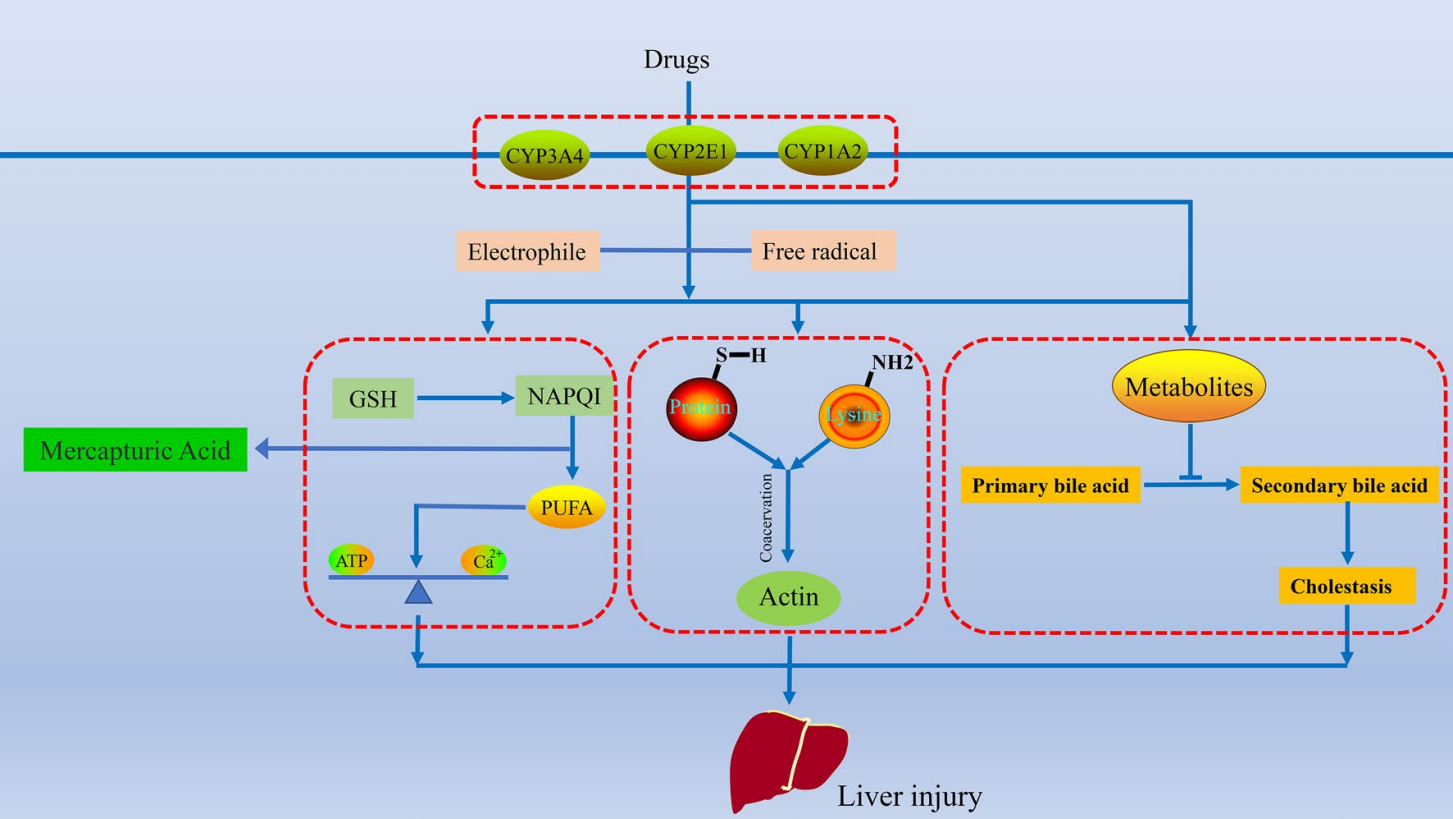
Possible mechanism of NSAIDs (example as APAP). The potential mechanisms involved are CYP450 metabolites interact with cellular macromolecules, destroy protein structure, lipid peroxidation, destroy ion gradient, and disrupt calcium ion transport, inhibit ATP synthesis and bile acid synthesis

Acetaminophen (APAP) is an NSAID with definite hepatotoxicity in clinical applications. At present, the main cause of APAP-induced hepatotoxicity is its metabolite N-acetyl-p-benzoquinone imine (NAPQI). APAP is mainly metabolized into glucosinolates and sulfates. When treated with APAP, these compounds accounted for 80–90% of the total metabolites. A small amount of APAP is metabolized by CYP450 to produce an intermediate, and the intermediate generates NAPQI. NAPQI directly interacts with glutathione, which exerts a detoxification effect. When glutathione is depleted, hepatocytes are affected by toxic metabolites, causing cell necrosis [28–30]. APAP toxicity is mediated by nitric oxide (NO), which scavenges superoxide and produces peroxynitrite, resulting in protein nitration (3-tyrosine) and tissue damage [31, 32] (Fig. 2).

### Anti-epileptic drugs (AEDs)

The treatment of epilepsy requires the long-term use of anti-epileptic drugs. ADR may occur in this process, affecting the treatment and quality of life of patients and, in severe cases, even threatening their life. Carbamazepine, phenobarbital, valproic acid, phenytoin, lamotrigine, and felbamate are associated with DILI [33]. Recent studies have shown that liver injury caused by carbamazepine may be related to drug metabolism and mitochondrial dysfunction [34].

Under the action of the CYP450 enzyme, the metabolites of AEDs covalently bind to the macromolecular proteins in cells or directly peroxidate the unsaturated fatty acids on the hepatocyte membrane, resulting in reduced ATP levels. The dynamic imbalance of Ca2+ in the cells leads to the rupture of actin fibers near the hepatocyte surface and disruption of the vesicular structure of the cell membrane, and then the cells rupture and dissolve [35].

Another mechanism of AEDs is blocking the beta oxidation and respiratory chain functions of mitochondria, leading to the failure of normal metabolism of free fatty acids, increased anaerobic fermentation, the accumulation of lactic acid and mitochondrial ROS production. ROS oxidize liposomes, cause lipid peroxidation, alter the mitochondrial membrane permeability, induce mitochondrial DNA damage, and induce cytokine (such as TNF-α and IL-8) production, further promoting liver damage [36].

### Anti-fungal drugs

Ketoconazole (KT) is a typical antifungal drug, but its hepatotoxicity mechanism has not been resolved. Children and people over 60 years of age who take KT have liver toxicity incidences of 2.2% and 14%, respectively. In addition, people who take KT orally have a higher incidence (5.5%) of liver toxicity than specified in the drug instructions [37]. Some scholars have suggested that active metabolites may be the cause of liver toxicity. One reported metabolite of KT is N-deacetyl ketoconazole (DAK). DAK is further metabolized by flavin-containing monooxygenases (FMOs) into the potentially toxic molecule dialdehyde [38].

### Chinese herbal medicine

In recent years, the side effects of hepatotoxicity caused by Chinese herbal medicine have become increasingly prominent due to the increased use of Chinese medicine and have even become one of the main causes of DILI. According to reports, herb-induced liver injury (HILI) accounts for approximately 23.61% (34/144 cases) of all DILI cases [39]. A literature review found that the main Chinese herbal medicines that cause HILI include Polygonum multiflorum, Psoralen, Corydalis and Rhubarb [40], in which alkaloids and terpenoids are the main causes of hepatotoxicity [41]. The main reasons for the increase in HILI include the unqualified quality of traditional Chinese medicine, nonstandard clinical use, long-term treatment with large doses and improper compatibility, which lead to the aggravation of its toxic side effects [39]. HILI is not exclusively caused by Chinese herbal medicine itself. Rather, multidrug combinations, complex compositions, patient physique and other underlying diseases play a role in HILI.

HILI is mainly divided into two types: direct hepatotoxicity and idiosyncratic hepatotoxicity [42]. Direct hepatotoxicity (inherent DILI) is the direct damage caused by drugs and their metabolites to liver cells or mitochondria. It is relatively rare at present and is closely related to drug dosages. It is predictable, controllable and relatively safe [43]. Diosgenin, baicalin, saikosaponin D, and tetrandrine reduced the survival rate of L02 cells and increased AST activity. The levels of ALT, LDH and ALP increased in the cell culture medium. The mechanism of liver injury may be related to the activation of p38α [44]. In contrast, idiosyncratic hepatotoxicity (idiopathic DILI) is more common in the clinic and includes immune idiopathic liver injury or inflammatory idiopathic liver injury. This type of DILI has no correlation with the drug dose, is difficult to predict and progresses faster [45, 46]. One example is liver injury caused by Polygonum multiflorum (PM).

**Fig. 3:**
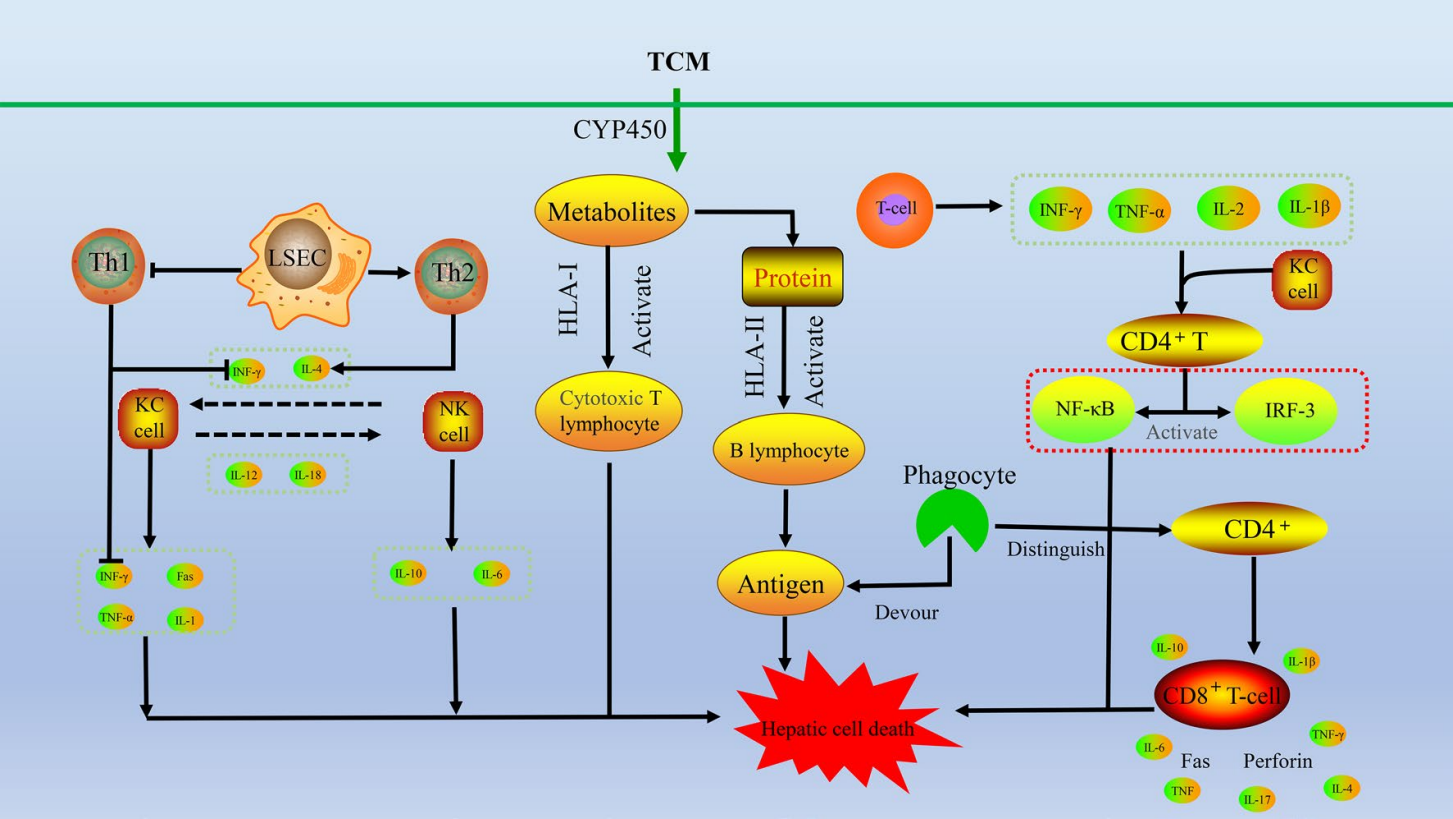
Possible mechanism of Immune idiosyncratic DILI. Drugs or their metabolic products combine with liver specific proteins to become antigens, which can be phagocytosed by phagocytes and expressed on the cell surface. They can be recognized by CD4^+^ cells, stimulate the production of cytokines, activate the CD8^+^T cells of effector cells, and produce cytotoxicity leading to liver damage

Cis-stilbene glucoside (cis-SG) is the main component of PM causing DILI. However, the dose of cis-SG leading to liver injury is greater than that used in medicinal materials, indicating that other components of PM may also be involved in inducing liver injury. The main form of stilbene glycoside in PM is trans stilbene glucoside (trans-SG), which exerts an immunomodulatory effect, combined with the key role of immune inflammation in PM-induced liver injury [47]. Idiopathic DILI induced by PM is the result of the synergistic actions of abnormal immune function, immune active components (trans-SG) and potential liver injury-susceptible components (cis-SG) in PM. When the body is in a hyperimmune state, the immune-promoting substances in PM (such as trans-SG) further enhance systemic immunity, increase the sensitivity of the liver to liver injury-susceptible components (such as cis-SG) in PM, and induce IDILI. He et al. [48] used an LPS model to study IDILI and found that compared with trans-SG alone, the liver injury caused by the combination of cis-SG and trans-SG at the same dose in medicinal materials was more serious. The same dose of trans-SG only slightly increased the immune response, indicating that trans-SG also played a role in PM-induced heterogeneous liver injury (Fig. 3).

### Other

Chlorpromazine and clozapine are commonly used first-line antipsychotic drugs that cause a certain degree of liver injury. Chlorpromazine directly induces liver toxicity, and clozapine increases the levels of transaminases in patients [49, 50]. Toxic free radicals are produced from antipsychotic drugs during liver metabolism, which directly inhibit Na+ - K+ − ATP enzyme activities in liver cells, thus reducing the stability of the liver cell membranes and detoxification enzymes and resulting in elevated transaminase levels and chronic liver damage [51].

### Bioactive components of natural drugs for liver protection

**Fig. 4:**
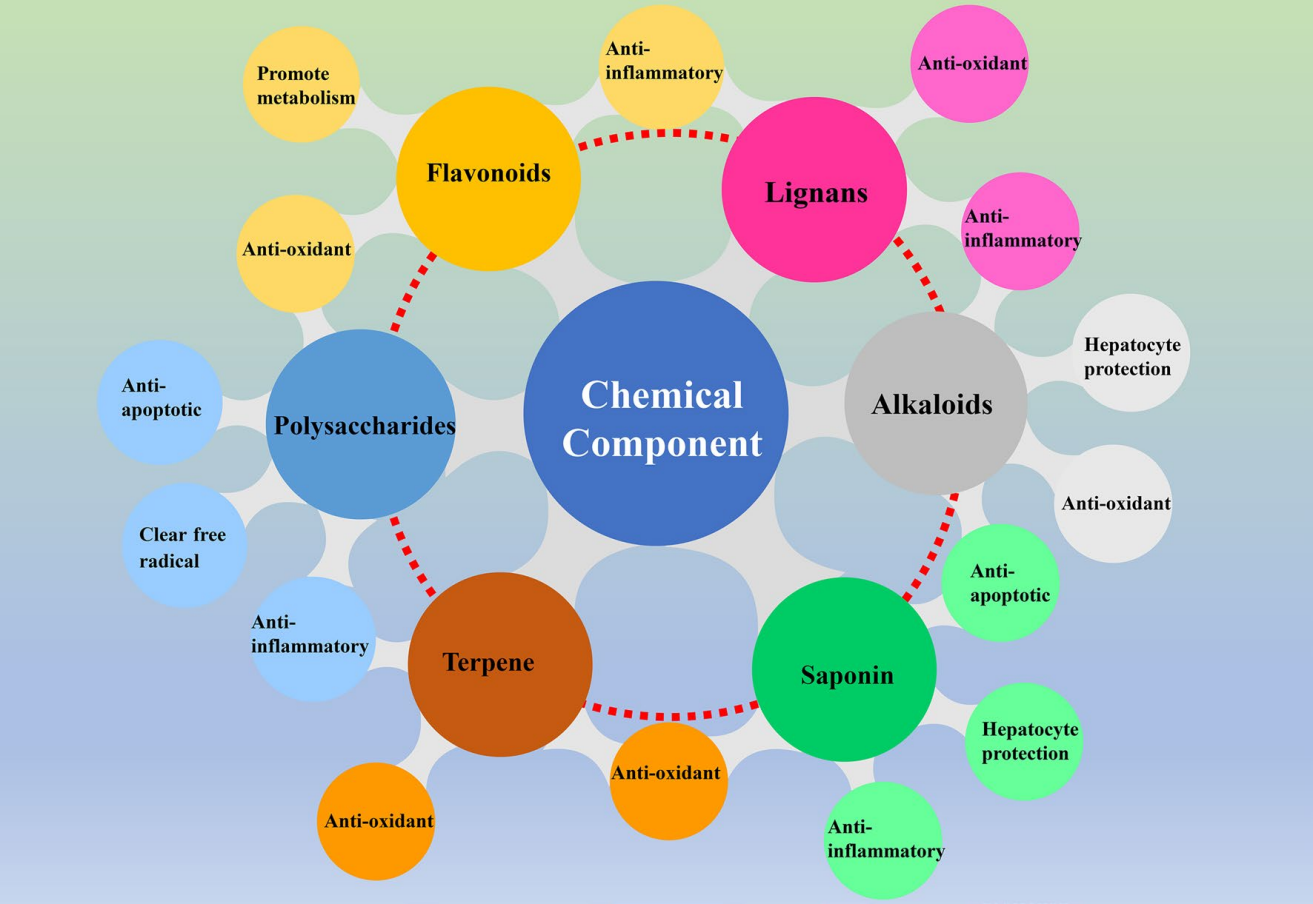
Bioactive components and mechanism of natural drugs for liver protection

A variety of chemical components in natural drugs have certain hepatoprotective activity. According to recent studies, flavonoids, polysaccharides, lignans, alkaloids, terpenes and other components have good hepatoprotective activity and good effects on drug-induced liver injury [52]. Their mechanisms of action are mainly to inhibit lipid peroxidation, promote the recovery of the liver cell membrane, eliminate oxygen free radicals, inhibit mitochondrial dysfunction, ameliorate cholestasis, and inhibit the secretion of inflammatory factors (Fig. 4).

### Flavonoids

Flavonoids are widely distributed in natural drugs and have good activity. They mainly includes flavone, flavonols, dihydroflavonoids and other related compounds. Most flavonoids exert anti-inflammatory, antioxidant and hepatoprotective effects.

#### Flavone

Chrysin protects against methotrexate (MTX)-induced hepatotoxicity by restoring cellular antioxidant defenses and downregulating the expression of p53, Bax and caspase 3 [53]. Flavonoids in Rosae Laevigatae Fructus also prevent DNA fragmentation and changes in the mitochondrial ultramicrosome system. The expression of TNF-α, Fas/FasL and the Burlington gene was significantly decreased, and the level of Bcl-2 was significantly increased in a previous study [54]. In addition, total flavonoids from Acacia and Propolis also exert protective effects on liver injury [55].

Baicalin protects against liver injury by inhibiting the activity of NF-κB and reducing TNF production, which may be related to the upregulation of HO-1 protein expression and activity [56].

#### Flavonol

Quercetin intervention effectively reduces the APAP-induced liver damage. Its mechanism is not only related to activating the Nrf2 signaling pathway and increasing the activity of antioxidant defenses but also to inhibiting the NF-κB signaling pathway, which is closely related to reducing the release of proinflammatory factors [57]. Quercetin also inhibits cisplatin-induced liver injury in mice by regulating the NLRP3 inflammasome pathway [58]. Hypericin increases the activity and mRNA expression of uridine diphosphate glucuronosyltransferases (UGTs) and sulfonyltransferases (sults), inhibits CYP2E1 activity, inhibits the formation of toxic intermediates and promotes the detoxification of APAP in the liver [59].

#### Dihydroflavone

Hesperidin reduces the infiltration of inflammatory cells and the production of proinflammatory cytokines, blocks the activation of Toll-like receptor (TLR)-4 signaling, and reduces oxidative stress and inflammatory response induced by APAP in mice. Hesperidin inhibits APAP-mediated cytotoxicity, apoptosis and reactive oxygen species (ROS)-induced upregulation of heme oxygenase-1 (HO-1) mRNA and protein expression in mouse AML12 hepatocytes [60]. Naringin significantly increases the IC50 values of troglitazone and diclofenac sodium and reduces the expression of transaminase and caspase3. In addition, naringin prevents DILI by inhibiting the function of the CYP450 enzyme [61]. Josephine exerts an obvious protective effect on APAP-induced liver injury in mice by significantly decreasing the MDA level and significantly increasing SOD activity and the GSH level. Thus, its protective effect may be related to the inhibition of oxidative stress in the liver [62].

#### Other compounds

**Fig. 5:**
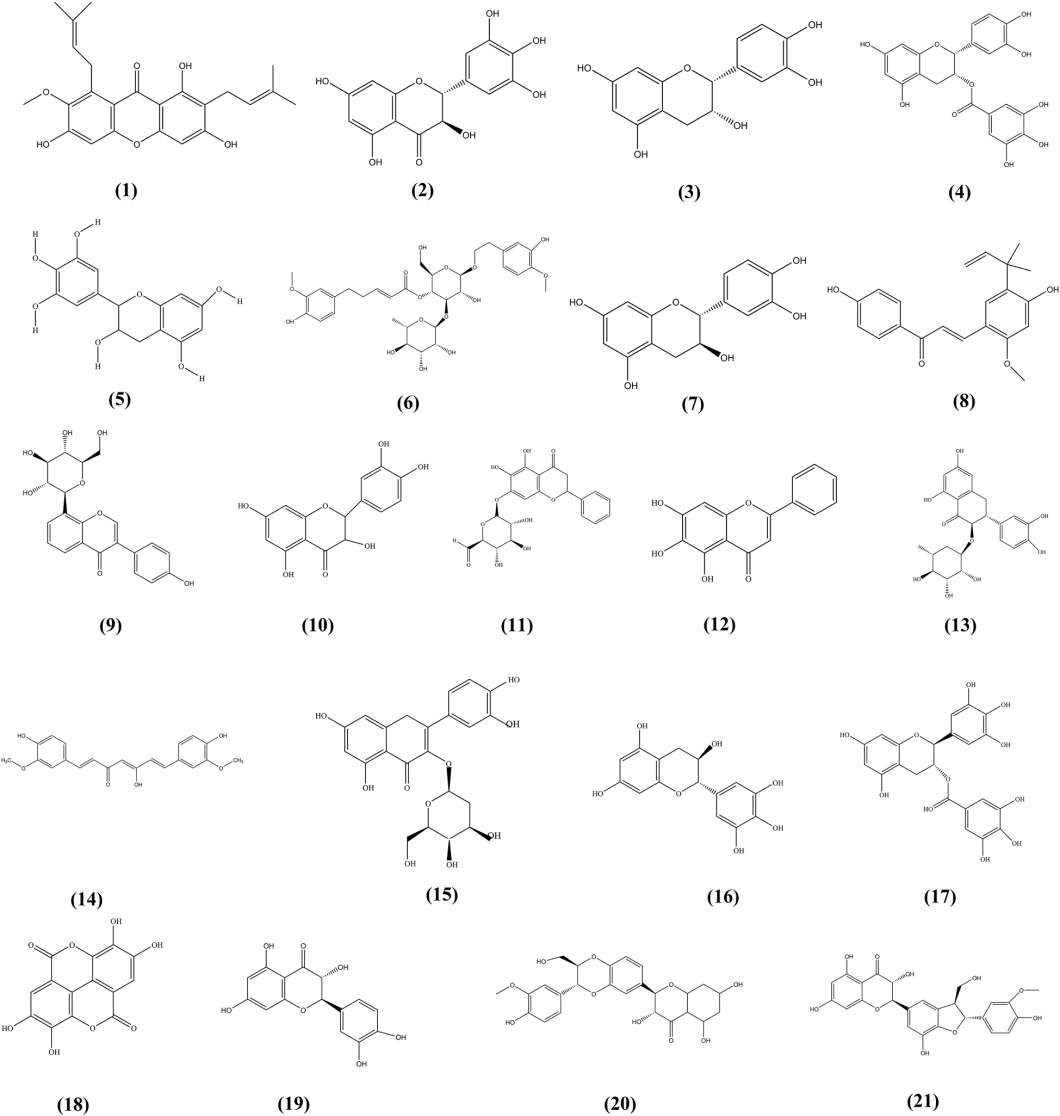
The chemical structures of flavonoids showing anti-DILI activity. (1) α-Mangostin; (2) Dihydromyricetin; (3) L-Epicatehin; (4) (-)-Epicatehin gallate; (5) (-)-epigallocatechin; (6) Martynoside; (7) (+)-Catechin; (8) Licochalcone A; (9) Puerarin; (10) Quercetin; (11) Baicalin; (12) Baicalein; (13) Astlbin; (14) Cuecumin; (15) Hyperoside; (16) (-)-Gallocatechin; (17) (−)-Gallocatechin gallate; (18) Ellagic acid; (19) (+)-Taxifolin; (20) Silymarin; (21) Silicristin

Cyanidin-3-O-β-glucoside (C3G) increases the expression of Gclc in the liver by increasing cAMP levels to activate protein kinase A (PKA) and then increases the phosphorylation of cAMP response element binding protein (CREB) to promote CREB-DNA binding and increase Gclc transcription. Increased expression of Gclc results in decreased ROS levels and proapoptotic signaling in the liver. In addition, C3G treatment reduces lipid peroxidation in the liver, inhibits the release of proinflammatory cytokines and prevents liver injury [63]. Silymarin is a flavonoid mixture with antioxidant, anti-inflammatory, immunoregulatory, antiproliferative, antiviral and antifibrotic activities. It exerts a good protective effect on liver injury caused by antituberculosis drugs and acetaminophen [64]. Flavonoids with specific anti-DILI activity are shown in Table 1. Moreover, their chemical structures are presented in Fig. 5.

**Table 1:** Anti-DILI effect and mechanism of flavonoids in natural medicines

No.	Natural medicine	Bioactive components	Mechanisms	The species investigated	References
1	*Fructus Livistonae*	Total flavonoids	Decreased the level of AST and MDA, increase the level of GSH and SOD, and inhibit the expressions of iNOS protein and NT protein	Pre-clinical model: L02 cell	[65]
2	*Polygonum perfoliatum* L	Total flavonoids	Inhibition the Fas pathway and anti-inflammatory; Nrf2—ARE signal pathway	Pre-clinical model: Male SPF mice (6–8 weeks old, 18–22 g); Kunming mice (30 males and 30 females) weighing,18–22 g	[66, 67]
3	*Toona sinensis* (Juss.) M.Roem	Quercitrin	Inhibition of pro-inflammatory genes via the suppressions of JNK and p38 signaling Protects the mitochondria and anti-oxidative stress	Pre-clinical model: 30 male Balb/c mice (6 weeks old, 20-25 g); Male BALB/c mice (18–22 g)	[68, 69]
4	*Polygonum orientale* L	Isoorientin	Activating Nrf2 via the AMPK/Akt/GSK3β pathway	Pre-clinical model: Wild-type and Nrf2-/-(knockout) C57BL/6 mice	[70]
5	*Sedum sarmentosum* Bung	Total flavonoids, Isorhamnetin	Decrease the content of MDA, increase the content of GSH, increase the activity of SOD and GSH-Px activity, and lower the release of ALT and AST	Pre-clinical model: L02 cell	[71]
6	*Pelargonium hortorum*	Pelargonidin	Removing excessive ROS, reduce levels of necrosis, inflammation, and hepatocyte apoptosis	Pre-clinical model: Male C57BL/6 mice (8 weeks old)	[72]
7	*Scutellaria baicalensis* Georgi	Baicalin, Baicalein	ERK Signaling Pathway, anti-inflammatory, ERK1/2 and PKC,	Pre-clinical model: Adult male C57BL/6 (B6) mice (8–10 weeks old); SPS male C57BL/6 mice (16–20 g)	[73, 74]
8	*Glycyrrhiza uralensis* Fisch	Glycyrrhizic acid	Down-regulation of CYP2E1 expression and deactivation of HMGB1-TLR4 signal pathway	Male Balb/c mice(8–10 weeks old)	[75]
9	*Rosa laevigata* Michx	Total flavonoids	Increase the protein expressions of Procaspase-3, Procaspase-8, FasL, Prohibitin and Bcl-2, and markedly decrease the protein expressions of Fas, Bax, p53 and NF-κB p65	Pre-clinical model: Male Kunming mice, (18–22 g)	[76]
10	*Penthorum chinense* Pursh	Pinocembrin	Reduce the level of MDA and significant increases in SOD activity and GSH level. Inhibition of liver oxidative stress	Pre-clinical model: 50 male C57BL/6 J mice	[77]
11	*Silybum marianum* (L.) Gaertn	Silymarin	Lower GSSG content, lower HO-1 induction, alleviated nitrosative stress, decreased p-JNK activation. improve ALT, AST, BUN, SCr and tissue NO levels	Pre-clinical model: Male BALB/c mice; Female mice (8–10 weeks, 30–35 g); Male Wistar rats (150–180 g)	[78–80]
12	*Hypericum monogynum* L	Hyperoside	Inhibition of the formation of toxic intermediates and promotes APAP liver detoxification	Pre-clinical model: Male Kunming mice (6 weeks old, 20–25 g)	[81]
13	*Garcinia mangostana*	α-otwistinin	Inhibiting oxidative stress and attenuates inflammatory response through NF-κB and MAPK signaling pathways	Pre-clinical model: Male ICR mice (4–5 weeks old, 20–25 g)	[82]
14	*Glycyrrhiza uralensis* Fisch	Licochalcone A	Protective effect of Nrf2-mediated oxidative stress on APAP-induced liver injury	Pre-clinical model: Nrf2 −/− (knockout) C57BL/6 mice	[83]
15	*Kaempferol galanga* L	Kaempferol	Antioxidative stress, promoting metabolism and inhibiting inflammation	Pre-clinical model: L02 cell	[84]
16	*Glycyrrhiza uralensis* Fisch	Monoammonium glycyrrhizin	Regulation of expression of hepatobiliary membrane transporters	Pre-clinical model: Male Wistar rats (180–220 g)	[85]
17	*Bacopa monnieri* (L.) Wettst	Bacoside	Enhance liver antioxidant capacity and reduce oxidative stress	Pre-clinical model: Female Wistar albino rats (170–200 g)	[86]
19	Crataegus pin.nati fida Bge *Curcuma longa* L	Quercetin and Curcumin	Mitigated the rise in TBARS and restored the activities of antioxidant enzymes. Protected liver histology, normalized liver and kidney functions	Pre-clinical model: Male Wistar rats (64, 200–235 g)	[87]
20	*Glycyrrhiza uralensis* Fisch	Glycyrrhizic acid	Inhibition of hepatocyte apoptosis induced by TNF-α	Pre-clinical model: Male C57BL/6 wild-type mice (6–8 weeks old)	[88]

### Polysaccharides

Polysaccharides are present in a wide range of animals and plants and are polymers composed of monosaccharides linked by glycosidic bonds. They participate in various life activities in organisms, mainly by inhibiting free radical-induced damage. They also regulate mitochondrial function and cytokines, inhibit the production of inflammatory mediators and prevent hepatocyte apoptosis [89–91].

In recent years, the ability of polysaccharides to improve the liver tissues of individuals with DILI has also been reported, including those that received APAP [92], antituberculosis drugs (isoniazid and rifampicin) [93], hormone drugs (hydrocortisone) [94] and antitumor drugs (cyclophosphamide, methotrexate, paclitaxel and cisplatin) [95–98]. Animal models have shown good results for candidate drugs in the prevention and treatment of DILI; new drug research and development continues to provide a reliable and feasible basis for DILI treatment.

Polysaccharides in Dendrobium officinale exert a hepatoprotective effect by inhibiting oxidative stress and activating the Nrf2-Keap1 signaling pathway [99]. Kunlun chrysanthemum polysaccharides significantly reduce the expression of caspase-3 proteins and Bax proteins and increase the expression of Bcl-2 proteins and the Bcl-2/Bax ratio. The mechanism may be related to its anti-inflammatory effects and the regulation of apoptosis-related protein expression [100]. Pachymaran increases the numbers of AKR7A-, c-Jun- and bcl-2-positive cells and decreases the number of Bax-labeled cells in the livers of mice with APAP-induced liver injury. The expression of NF-κB p65 and I Bα in liver cells is decreased in a dose-dependent manner [101].

Fucoidan inhibits CYP2E1 overexpression and reduces apoptosis caused by Bax, Bcl-2 and caspase-3. It also increases the antioxidant capacity (e.g., SOD), GSH-Px activity and GSH content in the liver. It reduces the levels of the inflammatory mediators TNF-α and IL-1β and the activity of iNOS; it also exerts good hepatoprotective effects [102]. SC polysaccharides significantly upregulate GSH expression in mice with liver injury, reduce MDA levels and upregulate the expression of Nrf2 to induce the expression of various detoxification enzymes and antioxidant genes (e.g., HO-1), thus alleviating oxidative stress and injury induced by DILI [103].

Polysaccharides with specific anti-DILI activity are shown in Table 2.

**Table 2:** Anti-DILI effect and mechanism of polysaccharides in natural medicines

No.	Natural medicine	Bioactive components	Mechanisms	The species investigated	References
1	*Fucus vesiculosus*	Polysaccharide	Inhibits the overexpression of CYP2E1 and reduces apoptosis caused by Bax, Bcl-2 and caspase-3. Anti-oxidant and anti-inflammatory	Pre-clinical model: Male SD rats(6-week-old)	[102]
2	*Schisandra chinensis* (Turcz) Baill	Polysaccharide	Up-regulate GSH and Nrf2, reduce the level of MDA. Anti-oxidant	Pre-clinical model: Male ICR mice (20–23 g)	[103]
3	*Artemisia argyi* Levl.et Vant	Polysaccharide	Increased blood glucose concentration indicators, hepatocyte protection	Pre-clinical model: Healthy adult rabbit	[104]
4	*Millettia pulchra Kurz var-laxior* (Dunn) Z.Wei	Polysaccharide	Attenuating free radical injury and inhibiting lipid peroxidation and lowering release of inflammatory factors; Reduce liver damage and activate the anti-oxidant defense system	Pre-clinical model: Healthy Kunming mice, half male and half male (18–20 g); Kunming SPF mice, half male and half male (18–20 g); Kunming SPF mice (18–20 g)	[105–107]
5	*Angelica sinensis* (Oliv.) Diels	Polysaccharide	Anti-oxidative	Pre-clinical model: Male ICR mice (35–40 g) and SD rats (120–150 g)	[92]
6	*Sophorae tonkinensis* Radix (S. tonkinensis)	Polysaccharide	Anti-oxidative	Pre-clinical model: Male ICR mice (25–30 g)	[108]
7	Sargassum	polysaccharide	Anti-oxidative, anti-inflammatory, and anti-apoptotic	Pre-clinical model: Male SD rats	[109]
8	*Sargassum polycystum*	Polysaccharide	Confirmed the effectiveness of the crude polysaccharide against acetaminophen-induced abnormality in rats	Pre-clinical model: Male albino Wistar rats (120–150 g)	[110]
9	*Aureobasidium* sp.	β-glucan	The increase of ALT and AST has a significant inhibitory effect	Pre-clinical model: Specific pathogen-free Balb/c mice (6–7 weeks)	[111]
10	*Sagittaria sagittifolia* L	Polysaccharides	Activation of NRF2 and its target antioxidant enzymes and inhibition of the expression of CYPs	Pre-clinical model: Sixty male BALB/c mice (18–22 g)	[112]
11	*Dicliptera Chinensis* (L.)Nees	Polysaccharide	Anti-inflammatory	Pre-clinical model: 60 SPF Kunming mice, (18–22 g), half male and half female	[113]
12	*Mangifera indica* L	Polysaccharide	Anti-oxidative	Pre-clinical model: Adult male albino Wistar rats (145–155 g)	[114]
13	*M. chinensis* Maxim cv. *jiangxiangru*	The Polysaccharide contains 11% uronic acid and 9% protein, and the polysaccharide part contains rhamnose, ara It is composed of primary sugar, mannose, glucose and galactose, and the molar ratio of monosaccharides is 5.3: 12.3: 3.4: 12.2: 32.6: 30.6	Anti-oxidative	Pre-clinical model: KM mice (8 weeks old, 20 ± 2 g)	[115]
14	*Polygonum Cillinerve* (Nakai) Ohwi	Polysaccharide	Anti-oxidative	Pre-clinical model: ICR mice (8 weeks old, 20 ± 2 g)	[116]
15	*Atractylodes macrocephala* Koidz	Polysaccharide	TLR4 signaling pathway	Pre-clinical model: Healthy 1-day-old Magang goslings	[117]
16	*Eucommia ulmoides* Oliv	Polysaccharide	Decline in ALT, AST, MDA, SOD values	Pre-clinical model: Kunming clean grade, 60 mice, weight (8 weeks old 21 ± 4) g, half male and half male	[118]
17	*Ostrea plicatula* Gmelin	Crude polysaccharide	Activation the Nrf2-ARE pathway and anti-oxidative stress	Pre-clinical model: Male BALB/c mice, (6 weeks old)	[119]
18	*Phellinus linteus*	*P*olysaccharide	Anti-oxidative	Pre-clinical model: Kunming female mice (5 weeks old, 22–30 g)	[120]
19	*Polygonatum sibiricum*	Crude polysaccharide	Anti-oxidative	Pre-clinical model: 40 clean-grade SD rats, half male and half male, (160–180 g)	[97]
20	*Cordyceps militaris* link	*P*olysaccharide	Anti-oxidative	Pre-clinical model: ICR male mice (30 ± 2 g)	[121]
21	Cordyceps cicadae	*P*olysaccharide	Anti-oxidative, scavenging free radicals	Pre-clinical model: Kunming mice (20 ± 2 g)	[122]
22	Sagittaria Sagittifolia L	*P*olysaccharide	Inhibition on CYP2E1 and CYP3A4	Pre-clinical model: Human HepG 2 cell	[123]
23	Pinus koraiensis Sieb. et Zucc	*P*olysaccharide	Improving hepatic antioxidant capacity via NRF2/ARE pathway and regulating inflammation response	Pre-clinical model: Mail SPF Kunming mice (20–22 g)	[124]
24	Seaweed	*P*olysaccharide	Up-regulate the expression of Ntcp	Pre-clinical model: Wistar male rat (200–220 g)	[125]
25	*Hippophae rhamnoides* Linn	*P*olysaccharide	Suppressed the expression of TLR4 and p-JNK	Pre-clinical model: Male C57BL/6 mice (8 weeks old)	[126]
26	*Astragalus membranaceus* var. mongholicus	*P*olysaccharide	Anti-oxidative	Pre-clinical model: SD SPF rat, grade, for both sexes (200–220 g)	[127]
27	*Panar japonicas* C.A. Mey	*N*eutral polysaccharide (70.61%); uronic acid (15.89%)	Anti-oxidative and anti-inflammatory	Pre-clinical model: Kunming SPF male mice (20–22 g)	[128]
28	*Dendrobium officinale* Kimura et Migo	*P*olysaccharide	Suppressing the oxidative stress and activating the Nrf2 − Keap1 signaling pathway	Pre-clinical model: Male ICR mice (6–8 weeks old)	[129]
29	*Inonotus obliquus*	Polysaccharide (98%)	Anti-oxidative	Pre-clinical model: 40 clean-grade mice (20–25 g)	[130]
30	*Poria cocos*	*P*olysaccharide	Suppressing inflammatory response and apoptosis in liver cells	Pre-clinical model: Male Kunming mice (6–7 weeks old, 18–22 g)	[131]
31	*Schisandra chinensis* (Turcz) Baill	*P*olysaccharide	Inhibition of hepatocyte apoptosis	Pre-clinical model: ICR male mice (19–22 g)	[132]
32	*Pleurotus citrinopileatus* Sing	*P*olysaccharide	Regulate activity of CYP2E1 and CYP3A	Pre-clinical model: KM mouse, half male and half female (20–22 g)	[133]
33	*Morus alba* L.	Total polysaccharides	Anti-oxidative and anti-inflammation	Pre-clinical model: Kunming SPF male (8 weeks old, 18–23 g)	[134]
34	*Atractylodes chinensis* (DC.) Koidz	*P*olysaccharide	Reduce transaminase activity, lower ALT, AST	Pre-clinical model: KM male mice (27–30 g)	[135]

### Alkaloids

Alkaloids are nitrogen-containing alkaline compounds that exist in the biosphere (mainly plants) and have significant biological activities. According to recent studies, many alkaloids exert protective effects on the liver. Berberine significantly reduces hepatic MDA and MPO levels, inhibits JNK phosphorylation and upregulates the expression of Nrf-2 in the nucleus and its downstream gene Mn-SOD. In addition, BBR pretreatment significantly reduces the expression of the proinflammatory cytokines HMGB1 and p-p65 and the cleavage of caspase-1 and inhibits the infiltration of macrophages and neutrophils, producing a significant preventive effect on DILI [136].

**Fig. 6:**
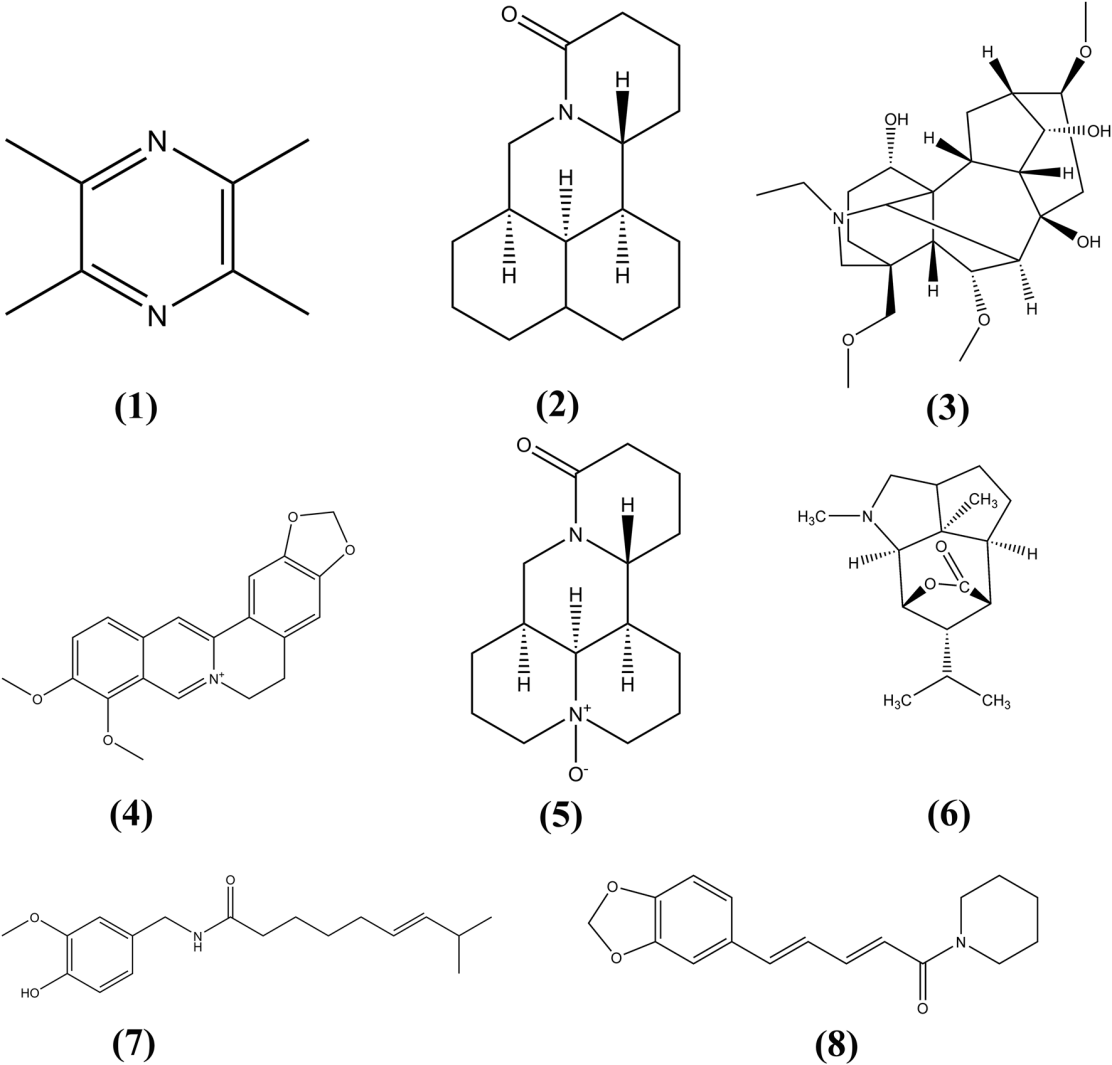
The chemical structures of Alkaloids showing anti-DILI activity. (1) Ligustrazine; (2) Matrine; (3) Aconine; (4) Berberine; (5) Oxymatrine; (6) Dendrobine; (7) Capsaicin; (8) Piperine

Aconitine protects hepatocytes from APAP-induced damage by inhibiting mitochondrial dysfunction. Aconitine significantly inhibits the APAP-induced decrease in glutathione levels. The mitochondrial membrane potential and Bcl-2-related protein levels are also decreased, but Bcl-2 and cytochrome-C levels in the mitochondria are increased. Thus, AC protects hepatocytes from APAP-induced toxicity by inhibiting mitochondrial dysfunction [137]. Ligustrazine exerts a protective effect on APAP-induced acute liver injury by inhibiting oxidative stress and inflammation. Its mechanism may be related to the regulation of NF-κB and MAPK signaling pathways [138]. Alkaloids with specific anti-DILI activity are shown in Table 3. Moreover, their chemical structures are presented in Fig. 6.

**Table 3:** Anti-DILI effect and mechanism of Alkaloid in natural medicines

No.	Natural medicine	Bioactive components	Mechanisms	The species investigated	References
1	*Coptis chinensis* Franch	Berberine	Inhibiting oxidative stress, hepatocyte necrosis and inflammatory response	Pre-clinical model: Male C57BL/6 mice (18–22 g)	[136]
2	*Aconitum carmichaelii* Debx	Aconine	Inhibiting mitochondrial dysfunction	Pre-clinical model: HepaRG cells	[137]
3	*Acanthi Ilicifolii Herba seu* Radix	Alkaloid A	Down regulating the expressions of NO and iNOS, and reducing the expression of protein p-ERRK1/2	Pre-clinical model: Kunming SPF male (18–22 g)	[139]
4	*Piper nigrum* L	Piperine	Antioxidant, anti-inflammatory, and anti-apoptotic	Pre-clinical model: Swiss mice (25–30 g)	[140]
5	*Capsicum annuum* L	Capsaicin	Inhibiting the inflammatory response, attenuating oxidative stress, and reducing hepatocyte apoptosis	Pre-clinical model: Healthy male Balb/c mice (6 weeks old, 18–20 g)	[141]
6	*Dendrobium nobile*	Dendrobine	Inhibiting miR-295-5p	Pre-clinical model: ICR (C57BL6) mice	[142]
7	*Nelumbo nucifera*	Total alkaloids	Activation of hepatic AMPK /Nrf2 cascade	Pre-clinical model: Kunming male (20–25 g)	[143]

### Saponin

Saponins are glycosides with a complex structure in the plant kingdom. Saponins are present in ginseng, diosgenin, licorice, notoginseng, Ophiopogon japonicus, *Platycodon grandiflorum*, Bupleurum and other natural medicines. Saponins display antitumor [144], hypoglycemic [145], cholesterol-lowering [146], hepatoprotective [147], immunoregulatory [148], anti-inflammatory [149] and other biological activities.

Ginsenoside Rk1 significantly reduces the serum levels of ALT, AST, TNF and IL-1β in mice with APA-induced liver injury. Ginsenoside Rk1 inhibits the activation of the apoptotic pathway by increasing Bcl-2 expression and reducing Bax protein expression levels, which significantly reverses APAP-induced liver necrosis and inflammatory cell infiltration [150].

Ginsenoside Rg1 significantly reduces APAP-induced hepatotoxicity and oxidative stress, reduces the expression of the Keap1 protein and upregulates the expression of GCLC, Ugt1a1, Sult2a1 and other genes. The Nrf2 signaling pathway is an important pathway mediating its hepatoprotective effect [151]. Saponins in *Platycodon grandiflorum* (PGSs) alter the phosphorylation of AMPK and PI3K/Akt, as well as the downstream signals, including the Bcl-2 family, caspase and NF-κB. The mechanism was mainly mediated by the NF-κB and AMPK/PI3K/Akt signaling pathways [152].

**Fig. 7:**
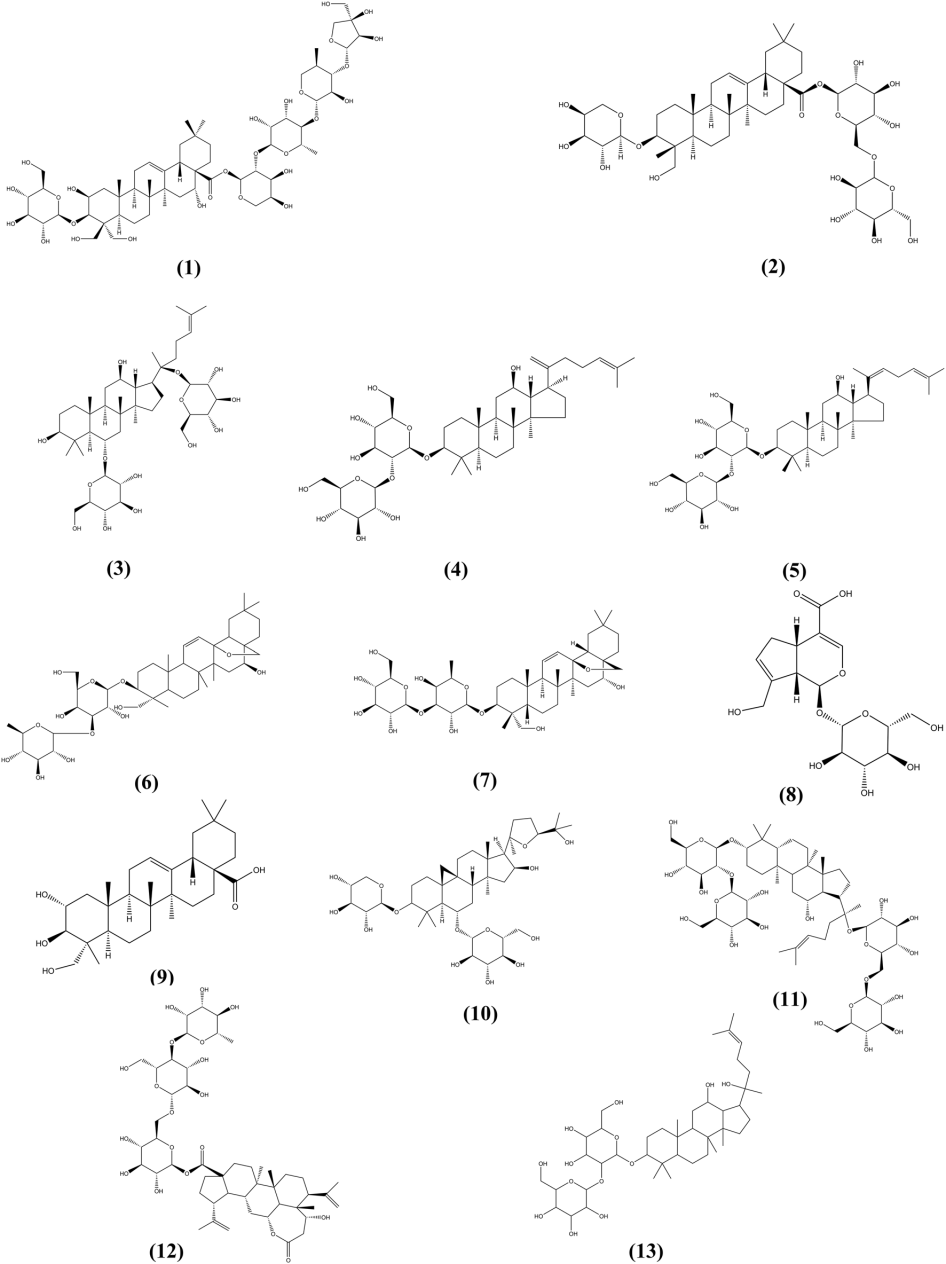
The chemical structures of Saponin showing anti-DILI activity. (1) Platycodin D; (2) Akebia saponin D; (3) Ginsenoside Rg1; (4) Ginsenoside Rg5; (5) Ginsenoside Rk1; (6) Saikosaponin A; (7) Saikosaponin D; (8) Geniposide; (9) Arjunolic acid; (10) Astragaloside IV; (11) Ginsenoside Rb1; (12) Chiisanoside; (13) Ginsenoside Rg3

Saponins with specific anti-DILI activity are shown in Table 4. Moreover, their chemical structures are presented in Fig. 7.

**Table 4:** Anti-DILI effect and mechanism of saponin in natural medicines

No.	Natural medicine	Bioactive components	Mechanisms	The species investigated	References
1	*Astragalus mongholicus*	Astragaloside IV	Anti-oxidative	Pre-clinical model: Male clean grade ICR mice, (18–22 g)	[153]
2	*Terminalia arjuna*	Arjunolic acid	Inhibition of P450-mediated APAP bioactivation and inhibition of JNK-mediated activation of mitochondrial permeabilization	Pre-clinical model: Male albino rats of the Wistar strain (4 weeks old, 120–130 g)	[154]
3	*Acanthopanax sessiliflorus*	Chiisanoside	Anti-inflammatory	Pre-clinical model: Mice	[138]
4	*Panax ginseng* CA Mey	Ginsenoside RK1	Anti-oxidative, anti-apoptosis, anti-inflammation and anti-nitrative effects	Pre-clinical model: Male ICR mice (8 weeks old, 22–25 g)	[150]
5	*Panax ginseng* CA Mey	Ginsenoside Rg1	Nrf2 signaling pathway	Pre-clinical model: Male C57BL/6 mice (8–10 weeks old, 20–25 g)	[155]
6	*Platycodon grandiflorum*	Platypodid D	NF‐κB and AMPK/PI3K/Akt signaling pathways	Pre-clinical model: Male ICR mice (8–10 weeks old, 20–22 g)	[156]
7	*Panax notoginseng* (*Burk.*) F. H. Chen	Total saponins	Anti-oxidative	Pre-clinical model: ICR SPF mouse, male, (18–25 g)	[157]
8	*Tribulus terrestris* L	Total saponins	Reducing Caspase-3 expression and inhibiting apoptosis of the liver cells	Pre-clinical model: Kunming mice (18–22 g)	[158]

### Lignans

Lignans are natural compounds synthesized by the polymerization of phenylpropanoids (C3–C6 monomers), which are widely distributed in various plants and have diverse structures and extensive biological activities. The lignans in *Schisandra chinensis* (SC) protect the liver and reduce serum ALT levels. For example, schisandrin methyl ester and its analogs are used to treat hepatitis in China [159]. SC lignans induce the hepatic expression of the PXR target genes Cyp3a11 and Ugt1a1, accelerate the metabolism of bile acid and increase the amount of bile acid flowing from liver to intestine or feces [160].

Gomisin N promotes liver sirtuin1 (SIRT1)-AMPK signaling, activates PPARα/PGC-1α, promotes fatty acid β oxidation, downregulates CYP2E1, upregulates antioxidant gene expression, inhibits inflammatory gene expression, and inhibits reactive oxygen species generation. In addition, GN prevents the decrease in SIRT1 signaling and AMPK phosphorylation to alleviate liver injury [161].

**Fig. 8:**
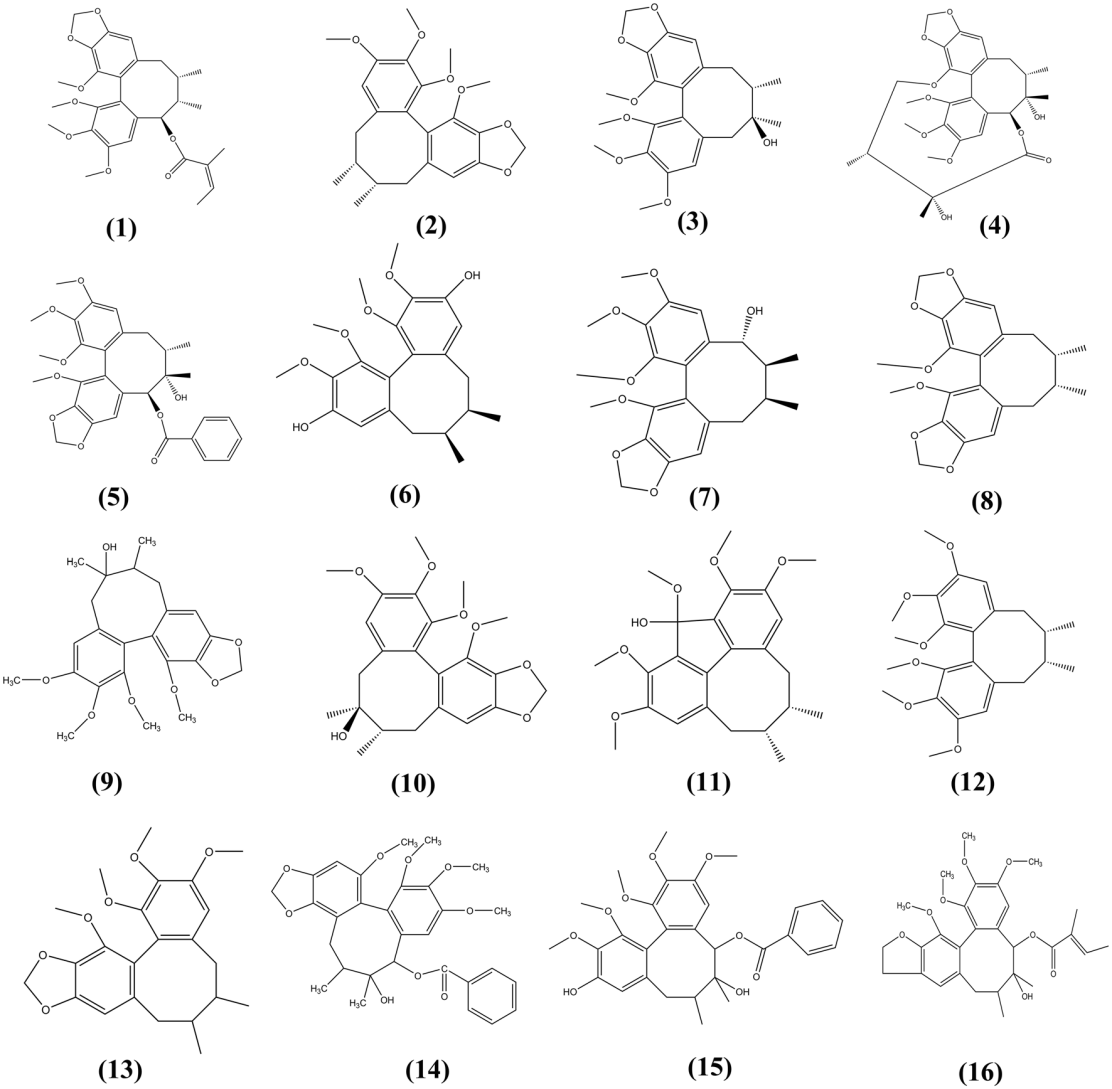
The chemical structures of lignans showing anti-DILI activity. (1) Angeloylgomisin O; (2) Gomisin N; (3) Schizandrol B; (4) Gomisin D; (5) Gomisin G; (6) Gomisin J; (7) Gomisin O; (8) Schisandrin C; (9) Schizandrol A; (10) Gomisin A; (11) Schisanhenol; (12) Schisandrin A; (13) Schizandrin B; (14) Schisantherin A; (15) Schisantherin E; (16) Schisantherin B

Lignans with specific anti-DILI activity are shown in Table 5. Moreover, their chemical structures are presented in Fig. 8.

**Table 5:** Anti-DILI effect and mechanism of lignans in natural medicines

No.	Natural medicine	Bioactive components	Mechanisms	The species investigated	References
1	*Mangnolia officinalis*	Magnolol	Anti-oxidative	Pre-clinical model: Male SD rat (200–250 g)	[162]
2	*Schisandra sphenanthera*	Lignans extract	Regulation of lipid metabolism	Pre-clinical model: Male C57BL/6 J mice (6 weeks, 18–20 g)	[163]
3	*Schisandra sphenanthera*	Schisandrol B	Attenuated the increases in ALT and AST activity, inhibiting the activities of CYP2E1 and CYP3A11. Abrogated APAP-induced activation of p53 and p21, and increased expression of liver regeneration and antiapoptotic-related proteins such as cyclin D1 (CCND1), PCNA, and BCL-2	Pre-clinical model: Male C57BL/6 mice (6–8 weeks old, 20–22 g)	[164]
4	*Schisandra fructus*	Schisandrin A, Schisandrin B, Schisandrin C, Schisandrol A, Schisandrol B, and Schisantherin A	Inhibited the enzymatic activities of three CYP450 isoforms (CYP2E1, CYP1A2, and CYP3A11) related to APAP bioactivation, and further decreased the formation of APAP toxic intermediate N-acetyl-p-benzoquinone imine (NAPQI) in mouse microsomal incubation system	Pre-clinical model: Male C57BL/6 mice (6–8 weeks old, 20–22 g)	[165]
5	*Schisandra fructus*	Schisandrin B	Induction of HSP27 and HSP70	Pre-clinical model: Male Institute of Cancer Research mice (18–22 g)	[166]

### Terpene

Terpenoids are natural compounds produced from isoprene or isoprane through various pathways. They are the main components of volatile oil and are widely distributed in nature. According to the number of isoprene units present in their structures, they are divided into monoterpenes, sesquiterpenes, diterpenes and triterpenes [167].

Citronella essential oil reduces the levels of ALT, AST, ALP, and MPO activity and NO production, inhibits the migration of neutrophils in mice with APAP-induced liver injury and shows antioxidant activity, thus ameliorating APAP-induced liver toxicity [168]. Astaxanthin inhibits the TNF-α-mediated JNK signaling pathway and the phosphorylation of ERK and P38, prevents ROS generation, inhibits oxidative stress, reduces hepatocyte necrosis, protects the liver and alleviates DILI [169]. Kamebakaurinecan significantly reduces ALT and AST levels and protects against DILI by inhibiting inflammation and oxidative stress [170].

**Fig. 9:**
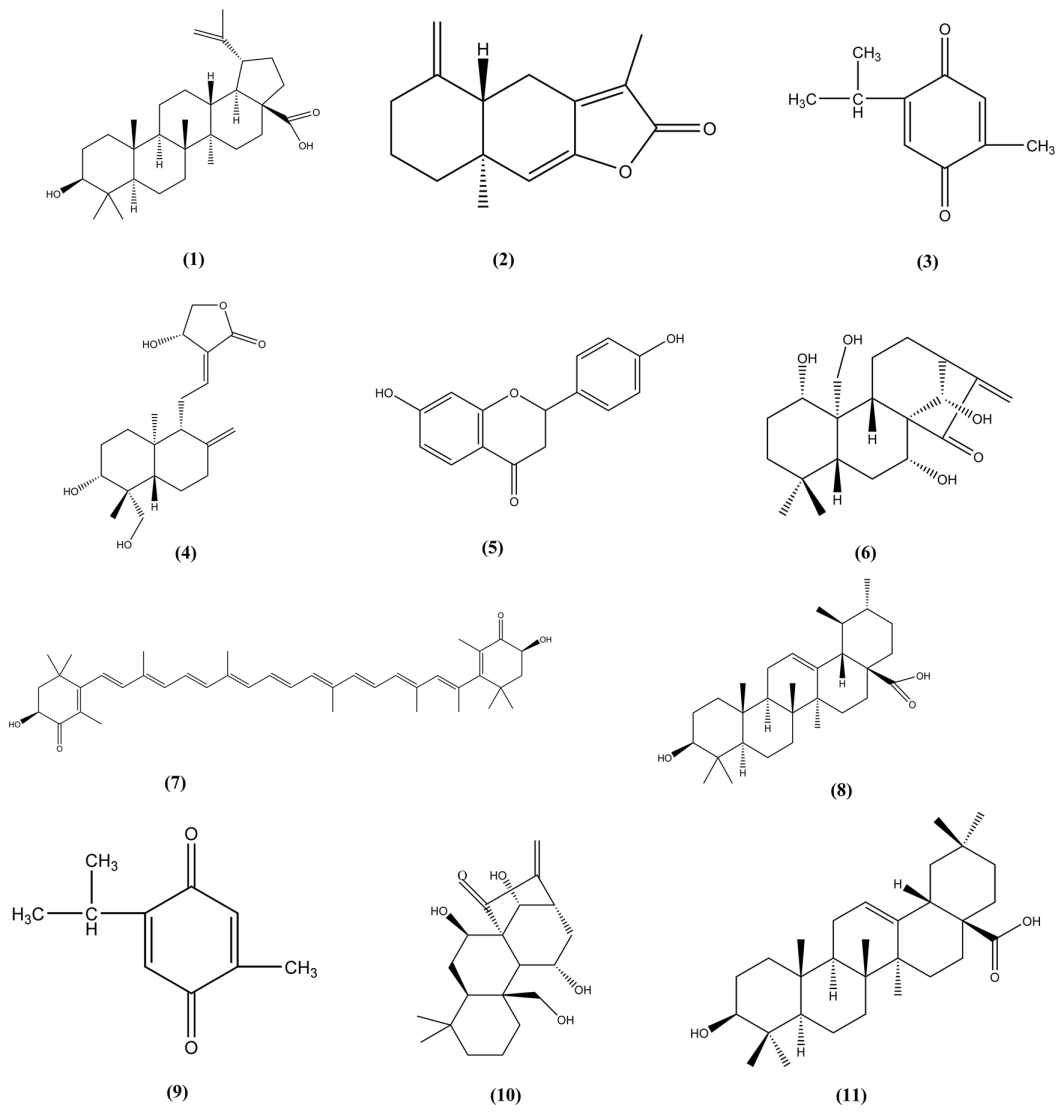
The chemical structures of terpene showing anti-DILI activity. (1) Betulinic acid; (2) Atracylenolide-1; (3) Thymoquinone; (4) Andrographis; (5) Liquiritigenin; (6) Kamebakaueine; (7) Ursolic; (8) Astaxanthin; (9) Thymoquinone; (10) Kamebakaurin; (11) Oleanolic acid

Terpenes with specific anti-DILI activity are shown in Table 6. Moreover, their chemical structures are presented in Fig. 9.

**Table 6:** Anti-DILI effect and mechanism of terpenes in natural medicines

No.	Natural medicine	Bioactive components	Mechanisms	The species investigated	References
1	Nigella sativa *Linn*	Thymoquinone	Decreases the level of protein and mRNA expression of α-SMA, collagen-I and the TIMP-1 to down-regulate TLR4 expression and significantly reducing the level of pro-inflammatory cytokines. Inhibits the phosphorylation of PI3K, enhances the phosphorylation of AMPK and LKB-1	Pre-clinical model: Male Kunming mice (6 weeks old; 18–23 g)	[171]
2	*Haematococcus pluvialis*	Astaxanthin	Alleviating hepatocyte necrosis, blocking ROS generation, inhibiting oxidative stress, and reducing apoptosis by inhibiting the JNK signaling pathway mediated by TNF-α and phosphorylation of ERK and P38	Pre-clinical model: male C57BL/6 mice	[169]
3	*Rabdosia excisa. KA*	Kamebakaurin	Inhibiting the inflammatory response and oxidative stress	Pre-clinical model: female C57BL/6 J mice (6 weeks old)	[170]
4	*Bouvardia ternifolia*	Ursolic acid and oleanolic acid	The levels of AST and ALT were significantly reduced	Pre-clinical model: male Balb/C mice (23–27 g)	[172]
5	*Bacopa monnieri*	Bacoside	Prevent lipid peroxidation and prevent free radicals	Pre-clinical model: female Wistar albino rats (170–200 g)	[173]

### Others

*Auricularia auricula* extract exerts antioxidant and protective effects on APAP-induced liver injury. The mitochondria-targeted antioxidant effects of chlorogenic acid may be one of its mechanisms [174]. Tovophylin A (TA) inhibits APAP-induced lipid peroxidation and improves the liver antioxidant capacity. It increases the mRNA expression of nuclear red blood cell-related factor 2 (Nrf2) and its target genes, inhibits the activation of nuclear factor-κB (NF-κB) and subsequently induces proinflammatory cytokine production to exert a significant protective effect on APAP-induced hepatotoxicity [175].

*Acalypha wilkesiana* extract significantly reduces TLR3 and TLR4 expression, thus activating MAPKs and NF-κB to reduce the levels of liver injury indicators and proinflammatory factors at the protein and gene levels to attenuate APAP-induced liver injury [176]. Tannic acid possesses antioxidant, anti-inflammatory and anti-apoptotic properties. Tannic acid inhibits excess IL-1β, TNF-α, c-fos, c-jun, NF-κB p65 and caspase-3 accumulation, inhibits Nrf2 and HO-1, and exerts significant hepatoprotective effects on APAP-induced hepatotoxicity [177]. Some polyphenols also reduce DILI by inhibiting the activities of the CYP2E1 and CYP1A2 enzymes [178]. Other bioactive components that inhibit DILI and have been identified to date are shown in Table 7.

**Table 7:** Anti-DILI effect and mechanism of extract for natural medicines

No.	Natural medicine	Bioactive components	Mechanisms	The species investigated	References
1	*Mucuna pruriens*	Ethanol extract	Anti-oxidative	Pre-clinical model: adult Wistar albino mice (15–20 g) and rats (100–200 g), either sex	[179]
2	*Solanum xanthocarpum* Fruit	50% ethanol extract	Anti-inflammatory, hepatoprotective activity	Pre-clinical model: wistar rats (150–170 g) and Swiss albino mice (25–30 g) of either sex	[180]
3	*Salvia miltiorrhiza*	Water extract	maintenance of mitochondrial metabolic activity, CYP2E1 inhibition, reduction of total glutathione depletion	Pre-clinical model: male SD rats (260–280 g)	[181]
4	*Moringa oleifera* Lam	Ethanol extract	Anti-oxidative	Pre-clinical model: male Wistar rats of (180–220 g)	[182]
5	*Pinellia ternate* (Thunb) Breit	Water extract	Anti-oxidative and regulate Nrf2	Pre-clinical model: male ICR mice (18–22 g)	[183]
6	*Garcinia mangostana* L.	Acetone extract	Activate Nrf2 and inhibit NF-κB signaling pathways	Pre-clinical model: male BALB/c mice (20–30 g)	[175]
7	*Schisandfa chinensis* (Turcz.) Baill	Extract	Active of Nrf2 signal pathway	Pre-clinical model: SPF SD rat, (160–200) g	[184]
8	*Phyllanthus emblica* L.	Extract	Active of Nrf2 /ARE signaling pathway	Pre-clinical model: male C57BL/6 J mice (5–8 weeks old, 18–22 g)	[185]
9	*Trapa natans*	50% ethanolic extract	Normalizes the altered liver marker enzymes and antioxidant defense status	Pre-clinical model: adult Wistar male rats (140–180 g)	[186]
10	*Auricularia delicata* (Fr.) Henn	Hexane, chloroform, ethyl acetate, and methanol extract	Anti-oxidative	Pre-clinical model: male rat	[187]

## The clinical application of natural drugs

In clinical practice, the use of single medications to treat DILI is infrequent (i.e., mainly for natural drug combinations), but their use for the treatment of antituberculosis drug-induced liver injury is relatively more frequent. This research is summarized in Table 8. Natural drugs and their combinations used to treat DILI were preliminarily investigated. It was found that the use frequency of natural drugs (e.g., SC, *Glycyrrhiza uralensis* and *Artemisia capillaris*) was high, with great exploration potential and application prospects.

**Table 8:** Clinical research

No.	Drug name	Natural medicine	Subject	Study design	Intervention	Length	Outcome	Quality of evidence	References
1	Shuganning injection	Yinchen, Ganoderma lucidum, Gardenia, Radix Isatidis, Scutellaria	60 patients (31 men and 29 women) with anti-tuberculous	Randomized, Controlled study	250 mL 10% glucose solution or 250 mL 0.9% NaCl with 6 mL Shuganing injection, 1 times/day	3 weeks	AST, ALT and TBIL and adverse reactions were lowered by treat group compared to control group. Effective rate was higher than control group	Ib	[192]
2	Sini Shugan decoction	Bupleurum, Tangerine Peel, Codonopsis, Cyperus rotundus, Red White Peony, Licorice, Poria, Rehmannia, Angelica, Atractylodes, Citrus aurantium	66 People (33 men and 33 women)	Randomized, Controlled study	Magnesium Isoglycyrrhizinate Injection (150 mg/times, 3 times per day) or Compound Glycyrrhizin Tablets (50 mg/times, 3 times/day)	/	AST, ALT, TBIL are significantly lower than before treatment	Ib	[193]
3	Sunflower hugan tablets	Bupleurum, Yinchen, Radix Isatidis, Schisandra, Pork Gallbladder Powder, Mung Bean	97 People (57 men and 40 women)	Randomized, Controlled study	Bicyclol (1 tablets /times, 3 times/day) Sunflower hugan tablets (4 tablets /times, 3 times/day)	4 weeks	Significantly reduce the incidence of adverse reactions, and the economy is better	Ib	[194]
4	Jiangmeiling capsule	Schisandrae Chinensis Fructus	63 People (42 men and 21 women)	/	Jiangmeiling capsule (3 tablets /times, 3 times/day) Inosine Tabletes (3 tablets /times, 3 times/day)	4 weeks	Liver function indexes returned to normal after switching to medical liver protection treatment	Ib	[195]
5	Shuganning	Yinchen, Ganoderma lucidum, Gardenia, Radix Isatidis, Scutellaria	164 People (101 men and 63 women)	Randomized, Controlled study	250 mL 10% glucose solution or 250 mL 0.9% NaCl with 6 mL Shuganing injection, 1 times/day	3 weeks	ALT, AST, TBiL are significantly lower than before treatment	Ib	[196]
6	Baidan Shugan prescription	Bupleurum, Cyperus rotundus, White Peony, Angelica, Dan Ginseng, Turmeric, Yinchen, Rhubarb, Whole Cucumber, Magnolia, Hawthorn, Gallus gallus, Astragalus, Atractylodes, Lily, Adenophora, Gentiana	196 People (102 men and 94 women)	Randomized, Controlled study	C: Diammonium glycyrrhizinate enteric-coated capsules (150 mg, 3 times/ day); T: Baidan Shugan prescription	4 weeks	The total effective rate of the test group were better than control group, ALT, AST, and TBiL significant improvement	Ib	[197]
7	Hugan Jiedu recipe	Bupleurum, Atractylodes macrocephala, Yinchen, Coptis chinensis, Guang turmeric, Weeping pot grass, Ginseng leaves	85 People (35 men and 50 women)	Randomized, Controlled study	Polyene Phosphatidyl choline (2 tablets /times, 3 times/day) Hugan Jiedu recipe (250 mL/times, 2 times/day)	6 weeks	ALT, AST, TNF-α, IL-6 values before and after treatment in the treatment group were statistically ignificant. The cure rate of the treatment group was significantly different than that of the control group	Ib	[198]
8	Yinlan Yigan Granule	Yinchen, forsythia, turmeric, isatis root, salvia, dangshen, angelica	69 People (43 men and 26 women)	Randomized, Controlled study	C: Silybin methylamine tablets (100 mg, 3 times/day) T: Yilanyigan Granules (9 g/times, 3 times/day)	2 months	The treatment effect of the observation group was significantly higher than that of the control	Ib	[199]
9	Liuwei Wuling tablets	Schisandra, Ligustrum lucidum, Forsythia, Zedoary turmeric, Cocory, Ganoderma lucidum spore powder	65 People (50 men and 15 women)	Randomized, Controlled study	C: Ganlixin capsule (100 mg /times, 3 times/day); T: Liuwei Wuling tablets (1.5 g/times, 3 times/day)	2 weeks	Total effective rate and TBIL of the treatment and control group were significant differences	Ib	[200]
10	Shuganning injection	Yinchen, Ganoderma lucidum, Gardenia, Radix Isatidis, Scutellaria	46 People (29 men and 17 women)	Randomized, Controlled study	C: Hepatic glycosides, vitamin C T: 250 mL 5% glucose solution with 20 mL Shuganing injection, 1 times/day	10 days	The serum level of ALT in treatment group was obviously lower than that of control group,	Ib	[201]
11	Compound Glycyrrhizin tablets	Glycyrrhiza	100 People (54 men and 46 women)	Randomized, Controlled study	T: Compound Glycyrrhizin tablets (50 mg/times, 3 times/day)	2 month	Compound Glycyrrhizin tablets can better improve the levels of ALT, AST, TBIL andγ-GT in patients with elevated transaminase induced by antipsychotic drugs than Glucurolactone tablets	Ib	[202]
12	Silibinin	Milk Thistle	568 patients were included with 277 in experiment group and 291 in control group	Prospective, multi-center, randomized, open-label and controlled trial	T: 2HREZ (S)/4HR and Silibinin, include isoniazid (H), 0.3 g/time, once a day; rifampin, 600 mg/time for patients weighted 50 kg, or 450 mg/time for patients weighted < 50 kg, once a day,; pyrazinamide (Z), 0.5 g/time, 3 times/day, ethambutol (E), 1.0 g/time for patients weighted 50 kg, or 0.75 g/ time for patients weighted < 50 kg, once a day; streptomycin (S), intramuscular injection of 0.75 g, once a day. Silibinin phospholipid complex capsules (35 mg/capsule,) were orally administered two capsules (70 mg) a time, with three times daily (210 mg/ day) C: 2HREZ (S)/4HR	8 weeks	ALT, AST, AKP, TBiL and DBiL. Liver injury symptoms included fatigue, anorexia, nausea, vomiting and abdominal distension; Other clinical outcomes were assessed based on improvement of clinical symptoms, acteriological results of sputum culture after 8 weeks of treatment and imaging analysis result	Ia	[203]
13	Silymarin	Silybum marianum (L.) Gaertn	55 People (22 men and 33 women)	Randomized, double-blinded, Controlled study	One tablet of silymarin (140 mg) or placebo was taken three times a day along with antituberculosis drugs. Study subjects were emphasized to make records when taking anti-tuberculosis and the study drugs	4 weeks	ALT, SOD, MDA and AOE; adverse events (i.e., decreased appetite, fatigue, confusion etc.) were reviewed from direct questioning and self-recording on the follow-up days	Ia	[204]
14	Silymarin	Silybum marianum	70 cases (37 men and 33 women)	Randomized Double blind	Group one was received Silymarin three times per day for two weeks. Each 140 tablet contains dried extract of Sylibum arianum equivalent to 140 mg Silymarin. The second group was received placebo with the same shape, size and dose intervals manufactured by the same company. Drugs and placebo were encoded	2 weeks	Liver function was being evaluated at the beginning of treatment and three times per week for 2 weeks by measurement of serum aspartate AST, ALT and TBIL. The patients were strictly monitored for drug induced adverse effects including nausea, vomiting, diarrhea, vertigo, exanthema and other allergic phenomenon	Ia	[205]
15	S. marianum capsule	S. marianum	370 cases (274 men and 96 women)	Randomized, Controlled trial	T: Received the standard anti-tuberculosis therapy plus the S. marianum capsule (oral, 200 mg, twice a day) C: Received the standard anti-tuberculosis therapy plus a vitamin C tablet	8 weeks	ATLI, the peak AST/ALT ratio, and the maximum altered ALP or GGT value. Secondary outcome measures included the occurrence of adverse drug reactions, prolonged treatment duration, taking second-line drugs, and the clearance of tuberculosis bacteria from the sputum after 2 months of treatment	Ia	[206]
16	Silymarin	Silybum marianum	103 cases (68 men and 35 women)	Double-blinded randomized controlled trial	Silymarin or placebo (with similar appearance with the study drug) were assigned to the study patient on the first day of anti-TB treatment. One tablet of silymarin (140 mg) or placebo was taken twice a day along with anti-TB drugs. The remaining tablets were counted on the days of follow-up to check patient’s compliance and adherence	8 weeks	The primary outcome of the study was to compare the development of anti-TB treatment related DILI defined by serum AST or ALT > 3 × upper normal limit (UNL) or TBil (TB) > 2 × UNL	Ia	[207]

Ia, randomized and controlled studies; Ib, evidence from at least one randomized study with a control group; IIa, evidence from at least one well-performed study with a control group; IIb, evidence from at least one well-performed quasi-experimental study; III, evidence from well-performed nonexperimental descriptive studies as well as comparative studies, correlation studies and case- studies; and IV, evidence from expert committee reports or appraisals and/or clinical experiences by prominent authorities

The authors further systematically reviewed SC and found that SC is a natural drug with great potential for the treatment of DILI. Its active components significantly improve the liver function of patients and exert significant therapeutic effects on DILI, with few reported adverse reactions. Thus, SC may be a very important and effective treatment from DILI. As many as 10 related preparations based on SC or its bioactive components have been widely used in the clinical treatment of elevated transaminase levels caused by various liver diseases. Examples include bicyclol, bifendate, gandening, Wuzhi capsules and Liuwei Wuling tablets.

The researchers studied 72 patients with liver injury caused by antituberculosis drugs. According to different methods of treating liver injury, the patients were divided into 37 patients in the observation group and 35 patients in the control group. The control group was treated with diammonium glycyrrhizinate, and the observation group was treated with bicyclol tablets. The total clinical effective rate of the observation group was significantly higher than that of the control group. After treatment, the levels of ALT, AST and TBIL in the two groups were lower than those before treatment, and the levels in the observation group were lower than those in the control group. Both treatments exert a good hepatoprotective effect, but the effect of bicyclol is better than diammonium glycyrrhizinate [188]. Liu observed the clinical efficacy of Liuwei Wuling tablets in preventing DILI caused by anti-TB drugs. Liuwei Wuling tablets were administered to the treatment group, and glucuronolactone tablets were administered to the control group. The occurrence of liver injury in the two groups was observed. One patient in the treatment group was diagnosed with liver injury and four patients in the control group had liver injury, with incidence rates of 6.7% and 26.7%, respectively, indicating that Liuwei Wuling tablets effectively prevent drug-induced liver injury caused by anti-TB drugs [189].

The mechanism by which Liuwei Wuling tablets treat DILI may be attributed to their anti-inflammatory and antioxidant properties. Liuwei Wuling tablets inhibit the expression of the inflammatory factors high mobility group protein 1 (HMGB1), TNF-α and IL-1β, increases GSH and SOD activities, and reduces MDA and triglyceride (TG) levels in the liver tissue [190]. In addition, Liuwei Wuling tablets inhibit the increase in the Th1 cell ratio and promote an increase in the Th2 cell ratio in mice to restore the Th1/Th2 balance and reduce liver injury [191].

## Limitations and future prospects

The purpose of establishing the control group in a clinical trial is to judge whether the changes observed in subjects before and after treatment are caused by the test drug or other factors. Randomized controlled trials are the most rigorous and reliable scientific method to evaluate the effects of medical intervention measures. The scientific and reasonable establishment of the control group is one of the most important steps in the design of clinical research, which is directly related to the results and conclusions of the research.

Natural drugs may exert a protective effect on DILI by inhibiting lipid peroxidation, promoting the recovery of the liver cell membrane, eliminating oxygen free radicals, inhibiting mitochondrial dysfunction, ameliorating cholestasis, restoring a balanced Ca^2+^ concentration and inhibiting the secretion of inflammatory factors. Due to the diversity of chemical components in natural drugs, they have many targets in the treatment of diseases. The same component may have multiple targets, and both common and different targets have been identified among different components. The components exert both independent and synergistic effects. Limited clinical trials investigating these drugs have mainly used natural drug combinations. Although the curative effect is accurate, the specific drugs and their mechanisms of action are difficult to explain. At present, most drugs have been analyzed in animal experiments and require further verification. Nevertheless, they still have a certain reference value.

## Conclusion

Natural drugs are a natural treasure worth developing and utilizing as new drugs. Drug development with natural drugs as the main raw material will be an important method to treat DILI in the future. We hope that this review provides a scientific basis for understanding existing products and prescriptions, further clarifies market positioning and provides a reference for the development of drugs for DILI.

## Data Availability

All data are available in the manuscript and they are showed in figures and tables.

## Abbreviations

ALR: Aug-menter of liver regeneration
ATP: Adenosine triphosphate
AMPK: Amp-dependent protein kinase
ALT: Alanine aminotransferase
AST: Aspartate aminotransferase
ARE: Antioxidant responsive element
ADR: Adverse drug reaction
ATDILI: Anti-tuberculosis DILI
APAP: Acetaminophen
AMPK/Nrf2: Adenosine 5 ‘-monophosphate (AMP)-activated protein kinase/nuclear respiratory factor2
BCL-2: B-cell lymphoma-2
CYP3A: Cytochrome P450 3A
CD1: Cyclin D1
DILI: Drug-induced liver injury
ERK: Extracellular regulated protein kinases
GSH: Glutathione
GSH-Px: Glutathione peroxidase
HSP27: Heat shock protein 27
HSP70: Heat shock protein 70
iNOS: Inductible Nitric Oxide Synthase
DHM: Dihydromyricetin
HO-1: Heme oxygenase-1
HILI: Herb-induced liver injury
INH: Isoniazid
IL-1β: Interleukin-1
IL-6: Interleukin-6
JNK: C-Jun N-terminal kinase
NSAIDs: LKB, liver kinase B
KT: Ketoconazole Non-steroidal anti-inflammatory drugs
MTX: Methotrexate
MDA: Malondialdehyde
NAPQI: N-acetyl-p-benzoquinoneimine
NRF2: Nuclear respiratory factor2
NF-κB: The master immune transcription factor nuclear factor kappa B
NO: Nitric Oxide
PI3K: Phosphoinositide 3-kinase
PKA: Protein kinase A
PCNA: Proliferating Cell Nuclear Antigen
PGSs: *Platycodon grandiflorum*
RFP: Rifampicin
PCNA: Proliferating cell nuclear antigen
ROS: Reactive oxygen species
SOD: Superoxide dismutase
SC: *Schisandra chinensis*
TNF: Tumour necrosis factor
TVX: Trovafloxacin
TNF-α: Tumor necrosis factor-alpha
TLR4: Toll-like receptors 4
TG: Triglyceride
